# Feasibility of RetinoQuest: e-health application to facilitate and improve additional care for retinoblastoma survivors

**DOI:** 10.1007/s11764-017-0642-z

**Published:** 2017-09-25

**Authors:** Nuray A. McNeill, Wijnanda A. Kors, Machteld I. Bosscha, Jennifer van Dijk, Armida W. M. Fabius, Ton Houffelaar, Irma M. Verdonck-de Leeuw, Annette C. Moll

**Affiliations:** 1 Department of Ophthalmology, VU University Medical Center, De Boelelaan 1117, 1081 HV Amsterdam, The Netherlands; 2 Department of Pediatric Oncology, VU University Medical Center, De Boelelaan 1117, 1081 HV Amsterdam, The Netherlands; 3 Department of Medical Psychology, VU University Medical Center, De Boelelaan 1117, 1081 HV Amsterdam, The Netherlands; 4 Department of Otolaryngology/Head & Neck Surgery, VU University Medical Center, De Boelelaan 1117, 1081 HV Amsterdam, The Netherlands; 5 Department of Clinical Psychology, VU University Medical Center, De Boelelaan 1117, 1081 HV Amsterdam, The Netherlands; 6 Department of Ophthalmology, VU University Medical Center, P.O. Box 7057, 1007 MB Amsterdam, The Netherlands

**Keywords:** Retinoblastoma, Quality of life, Patient reported outcomes, Clinical practice, E-health, Psychosocial

## Abstract

**Purpose:**

The current study aimed to evaluate the feasibility of RetinoQuest in clinical practice, from survivors and healthcare professionals’ (HCPs) point of view.

**Methods:**

RetinoQuest is a touch screen computer program to monitor health-related quality of life (HRQoL) of retinoblastoma survivors via patient-reported outcome measures (PROMs) targeting children (4–10 years) as evaluated by their parents (proxy measures), adolescents (11–18 years), and adults. Feasibility was evaluated by the actual time taken to complete the PROMs, acceptability of the time as perceived by the users, the content of PROMs in RetinoQuest, and overall satisfaction with RetinoQuest.

**Results:**

Ninety-six survivors participated: 41 parents of children, 38 adolescents, and 17 adults. Mean time to complete the evaluation form was 7.8 min (median 6.7, range 2.4–24.5), and 90% of the users stated that the time needed to complete PROMs in RetinoQuest was acceptable. The majority of users reported that it was important to answer the questions (88% of the parents, 66% of the adolescents, and 76% of the adult survivors) and that all important issues were covered, e.g., no missing questions (78, 84, and 76%, respectively). Satisfaction rate was high, 7.8 according to parents, 8.1 according to adolescents, and 7.7 for adults.

**Conclusions:**

RetinoQuest is a feasible e-health application to monitor HRQoL in retinoblastoma survivors in clinical practice.

**Implications for cancer survivors:**

This tool allows for open and structured communication which can lead to early detection of psychosocial impacts on quality of life and referral of the retinoblastoma survivors.

**Electronic supplementary material:**

The online version of this article (10.1007/s11764-017-0642-z) contains supplementary material, which is available to authorized users.

## Introduction

Retinoblastoma (Rb) is a malignant intraocular pediatric cancer. It occurs in the first years of childhood, usually between the ages of 0 and 5 years. The incidence is 1:17,000 newborns (10–12 new patients in the Netherlands each year) [1], which represents approximately 3% of all pediatric malignancies.

In spite of good survival chances, the psychosocial effects of Rb vary according to the severity of the disease and the treatment, late sequelae and coping mechanisms of the patient and parents [2]. Many survivors experience distress regarding their cosmetic appearance and the fear of blindness. Other concerns relate to recurrence of the disease, passing Rb to offspring, second primary tumors, restrictions in education and professional career, mobility, self-care, and relationships [3–8]. Furthermore, the parents’ ability to cope with the stress associated with diagnosis, treatment, and heredity impacts the psychosocial functioning and development of their affected child [2, 7, 9–12].

Van Dijk et al. showed that 30% of Rb survivors develop behavioral problems [8]. Adult survivors have 20% more psychological problems like anxiety or depression than a healthy reference group [3]. The study by Van Dijk et al. also noted that a history with Rb influenced the health-related quality of life (HRQoL) and daily living negatively in 50% of Rb survivors [3].

Though psychosocial problems are thus common among Rb survivors, they are infrequently discussed during the annual follow-up visits at the outpatient clinic. Adequate psychosocial care should include an initial survey of the psychosocial background and early detection of psychosocial problems. If necessary, survivors should be referred to one of the specialized psychosocial disciplines. In the past 10 years, interest in using patient-reported outcome measures (PROMs) to screen for psychosocial problems and the need for supportive care in routine clinical practice has increased [13–19]. Several studies have shown that using PROMs in clinical practice facilitates open communication [20, 21] regarding HRQoL between doctors and patients and offers the possibility of early detection of psychological problems [22, 23]. In the Netherlands, we already use validated questionnaires for cancer survivors such as OncoQuest, QLIC-ON PROfile, and KLIK [14, 15, 24]. However, these questionnaires do not highlight Rb-specific distress such as cosmetic appearance and the fear of blindness. Furthermore, OncoQuest is a HRQoL-monitoring computer-assisted system for adult cancer survivors, whereas our population consists of both adults and children. Thus, currently existing validated questionnaires are not fully applicable to monitor HRQoL of Rb survivors. Therefore, we decided to develop Rb-specific questionnaire which consists of a combination of OncoQuest software with questions from Strengths and Difficulties Questionnaire (SDQ) and General Health Questionnaire (GHQ) [25–28]. RetinoQuest is a touch screen computer program to monitor HRQoL of retinoblastoma survivors via three age-specific PROMs (Table 1). The objective of the present study was to evaluate the feasibility of RetinoQuest in clinical practice, from the survivors’ and health care professionals’ (HCPs’) point of view.

**Table 1:** Overview of Rb-specific questions in RetinoQuest

Parents (children 4–11 years)—13 items
How much distress did your child experience due to problems, complaints, and concerns
Do you think your child is limited by his/her vision during the daily activities
Are you aware of the support provided by the companies like Sensis, Visio or Bartimeus
Does your child have an artificial eye
Are there any problems with your child’s artificial eye
What type of education is your child currently in
Are you satisfied with the performance of your child at school
Do you think the (effects of) retinoblastoma affect the functioning of your child’s learning
Does your child get additional support for learning
If yes, does your child get this additional support at school
Did you get any information regarding heredity and retinoblastoma
If yes, who did give you this information
Do you have any questions regarding (the consequences of) retinoblastoma you would like to discuss with your doctor
Adolescents (11–18 years)—12 items
Are you limited in your daily activities considering your vision
Are you aware of the support provided by the companies Sensis, Visio or Bartimeus
Do you wear an artificial eye
Do you have any problems with your artificial eye
What type of education are you currently in
Are you satisfied with your performance at school
Do you have any difficulties at school considering your condition
Can you remove your prosthetic eye independently
Can you insert your prosthetic eye independently
Did you get any information regarding heredity and retinoblastoma
If yes, who did give you this information
Do you have any questions regarding your condition you would like to discuss with your doctor
Adults—10 items
Are you limited in your daily activities considering your vision
Are you aware of the support provided by the companies Sensis, Visio or Bartimeus
Do you wear an artificial eye
Do you have any problems with your artificial eye
What is your current occupation
Are you satisfied with your performance in your studies or work
Do you think the (effects of) retinoblastoma affect your performance in your studies or at work
Did you get any information regarding heredity and retinoblastoma
If yes, who did give you this information
Do you have any questions regarding your condition you would like to discuss with your doctor

## Methods and results

### Ethical consideration

All procedures performed involving human participants were in accordance with the ethical standards of the institutional and/or national research committee and with the 1964 Helsinki Declaration and its later amendments or comparable ethical standards (for further details, see online supplementary methods).

The current evaluation study was performed in agreement with recommendations of Dutch ethics committee with waiver of informed consent. Prior to the current study, survivors and their parents have been informed per letter regarding the use of RetinoQuest in the outpatient clinic to support ophthalmologic consultation. They were also informed that its use will be evaluated through an evaluation form. Apart from this, when filling out RetinoQuest, survivors and their parents are asked to give a consent to data analysis for scientific purposes by answering (yes/no) the following question in the software application: “The information obtained is also important for scientific research at our department. Your information will be processed anonymously and treated confidentially. Please indicate below if you give permission to use this information for research purposes.”

### RetinoQuest

**Fig. 1:**
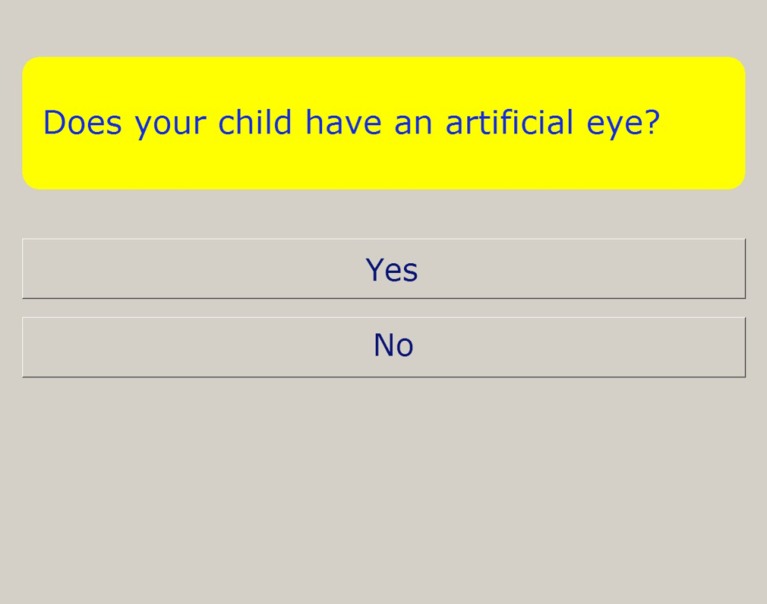
Example of questions presented on a full screen in RetinoQuest

**Fig. 2:**
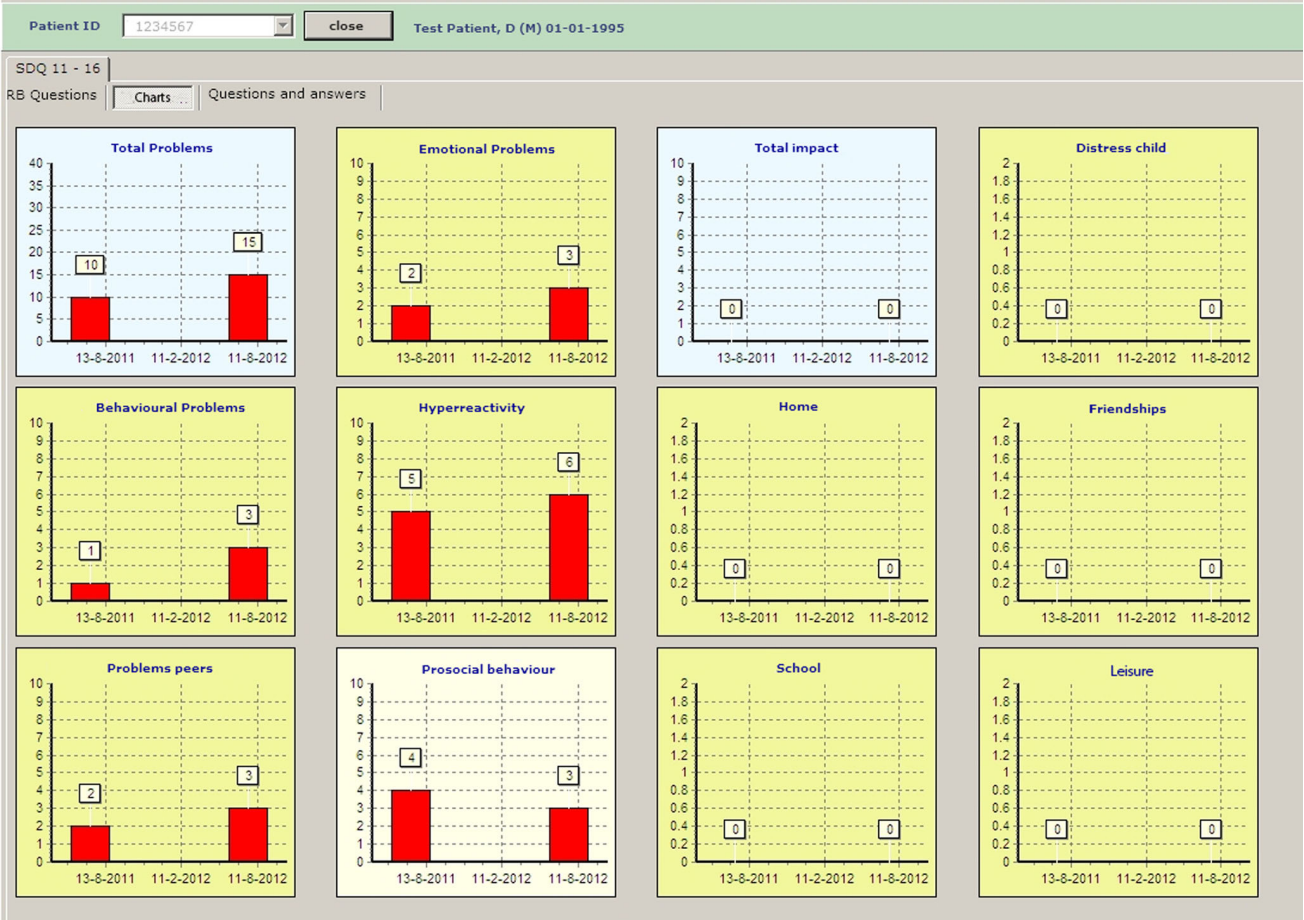
The results of the scores on consecutive visits schematically represented on a computer screen

RetinoQuest consists of the currently available validated questionnaires for oncological survivors in combination with Rb-specific questions. Dutch versions of SDQ 4-10 (parent proxy measure), SDQ 10-16, and GHQ-28 were used for the assessment of HRQoL [25–28]. The software program is written using Delphi2007 [14]. To meet the requirements of the visually impaired, the text is displayed in Verdana with a 24 to 28-point font size, in bright colors in boxes, and displayed on a 17-inch touch screen (Fig. 1). The data is processed in real time and is presented on the HCPs’ computer screen (Fig. 2) (for further details, see online supplementary methods).

### Procedures and analysis

For a period of 1 year, all eligible survivors and their parents, who visited the outpatient clinic, and two ophthalmologists were interviewed regarding the use of and the satisfaction with RetinoQuest at the end of each visit. Participants were assisted by a trained staff member or a researcher and were asked to complete an eight-item survey on feasibility of and satisfaction with RetinoQuest. The variables were measured via 10-point Likert scale. Time needed to complete RetinoQuest was logged by the computer program. Data was extracted from RetinoQuest, and frequencies and percentage frequencies were calculated for each variable. These frequencies are summarized in frequency table (Table 2). Ophthalmologists were asked to answer the question “Was the well-being profile discussed?” after each visit. In case of a negative answer, they were requested for an explanation, and in case of a positive answer, they were asked for the result of the discussion (e.g., advice or referral). Both ophthalmologists evaluated the use of RetinoQuest at the end of the study period through structured interviews (for further details, see online supplementary methods).

**Table 2:** Satisfaction rate of RetinoQuest according to participants

		Parents of patients (4–10 years), *n* = 41 (%)	Adolescents (11–18 years), *n* = 38 (%)	Adults, *n* = 17 (%)
Overall rating (scale 1–10)		7.8	8.2	7.8
Mean time to complete RetinoQuest		7.8 min	7.8 min	7.4 min
The program is easy to use	Strongly agree	40 (98%)	33 (89%)	14 (82%)
	Somewhat agree	1 (2%)	3 (7%)	1 (6%)
	Neutral	–	1 (2%)	–
	Somewhat disagree	–	1 (2%)	1 (6%)
	Strongly disagree	–	–	1 (6%)
Completing the questionnaire requires limited time	Strongly agree	40 (98%)	30 (79%)	16 (94%)
	Somewhat agree	1 (2%)	6 (17%)	1 (6%)
	Neutral	–	1 (2%)	–
	Somewhat disagree	–	–	–
	Strongly disagree	–	1 (2%)	–
The questions on the screen are clearly visible	Strongly agree	41 (100%)	37 (98%)	12 (71%)
	Somewhat agree	–	–	1 (6%)
	Neutral	–	1 (2%)	–
	Somewhat disagree	–	–	–
	Strongly disagree	–	–	4 (23%)
I understand the questions	Strongly agree	Not relevant	28 (74%)	Not relevant
	Somewhat agree		8 (21%)	
	Neutral		2 (5%)	
	Somewhat disagree		–	
	Strongly disagree		–	
It is important to complete the questionnaire	Strongly agree	36 (88%)	25 (66%)	13 (76%)
	Somewhat agree	3 (8%)	7 (19%)	1 (6%)
	Neutral	1 (2%)	4 (11%)	1 (6%)
	Somewhat disagree	1 (2%)	1 (2%)	2 (12%)
	Strongly disagree	–	1 (2%)	–
The questionnaire contains redundant questions	Strongly agree	1 (2%)	3 (8%)	2 (12%)
	Somewhat agree	1 (2%)	7 (18%)	7 (40%)
	Neutral	3 (7%)	3 (8%)	2 (12%)
	Somewhat disagree	3 (7%)	2 (3%)	2 (12%)
	Strongly disagree	33 (82%)	24 (63%)	4 (24%)
I miss some questions	Strongly agree	4 (10%)	1 (3%)	2 (12%)
	Somewhat agree	1 (2%)	2 (5%)	1 (6%)
	Neutral	4 (10%)	–	1 (6%)
	Somewhat disagree	–	3 (8%)	–
	Strongly disagree	32 (78%)	32 (84%)	13 (76%)
It is nice to complete the questionnaire at the hospital before the appointment	Strongly agree	34 (83%)	28 (74%)	13 (76%)
	Somewhat agree	4 (10%)	5 (13%)	2 (12%)
	Neutral	2 (5%)	5 (13%)	1 (6%)
	Somewhat disagree	–	–	–
	Strongly disagree	1 (2%)	–	1 (6%)

### RetinoQuest evaluation by participants

All 96 eligible survivors were enrolled in current study: 41 parents of child survivors, 38 adolescent survivors, and 17 adult survivors. The results are summarized in Table 2. Mean satisfaction rate was 7.8 (scale 1–10, median 8.0, range 1–10) among parent participants, 8.2 (scale 1–10, median 8.0, range 6–10) for adolescent participants, and 7.8 (scale 1–10, median 7.5, range 6–10) for adult participants. Mean overall satisfaction rate was 8.0 (median 7.8, range 1–10), and 78% of participants agreed with the statement “I would like to complete RetinoQuest at the outpatient clinic before the consultation.” Participants needed a mean time of 7.8 min (median 6.7, range 2.4–24.5) to complete the survey, and 90% of all participants stated that completing the survey takes limited time.

Ninety-eight percent of parent participants, 89% of adolescent participants, and 82% adult participants strongly agreed that the system is easy to use.

Eighty-eight percent of parent participants, 66% of adolescent participants, and 76% of adult participants strongly agreed that it is important to complete the questionnaire. Eighty-nine percent of parent participants, 66% of adolescent participants, and 36% percent strongly or somewhat disagreed that there were redundant questions. Four questions for the adolescent participants seemed redundant; all were derived from the SDQ questionnaire. The questions concerned the topics lying/cheating, fighting, helpfulness, and admiration by peers. Three adult participants suggested that they missed questions involving happiness/positive aspects of living with Rb, the past, and hereditary impact. Adult participants also indicated that there were too many questions about depression and suicide. Three parent participants indicated that they missed a question regarding the impact of Rb on the siblings of their affected child. Eighty-four percent of adolescent participants noted (strongly agreed) that no questions were missing. The majority of adolescent participants (74%) reported that the questions were clear.

Seventy-one percent of adult participants reported that the screen visibility was good. Three adult participants could not read the screen due to blindness/severe visual impairment. In these cases, the staff member assisted the participant by reading the text aloud.

### RetinoQuest evaluation by healthcare professionals

PROMs were discussed with 80% of the parent participants, 76% of the adolescent participants, and 76% of the adult participants during the consultations (Table 3). Main reasons for not discussing the PROMs were technical/logistic problems (11%) or due to a lack of a reason for discussion (profile normal/no questions in 10%). If discussed further, it resulted in clarification of the ongoing issues, answering of the questions, and if necessary advice (7%) or referral to psychologist (2%).

**Table 3:** Use of RetinoQuest by ophthalmologists

		Parents of patients (4–10 years), *n* = 41 (%)	Adolescents (11–18 years), *n* = 38 (%)	Adults, *n* = 17 (%)
Well-being profile discussed in detail during consultation?	Yes	33 (80%)	29 (76%)	13 (76%)
	No	8 (20%)	9 (24%)	4 (24%)
If not, reason	Well-being profile normal	4 (45%)	1 (11%)	1 (17%)
	Patient no questions	1 (11%)	1 (11%)	2 (50%)
	Known with these problems	–	1 (11%)	–
	Forgotten	–	1 (11%)	–
	RetinoQuest filled out after consultation	2 (22%)	3 (34%)	–
	Profile not available due to computer failure	2 (22%)	2 (22%)	2 (33%)
If yes, result	Issues have become clear	11 (33%)	14 (50%)	6 (50%)
	No problems	4 (14%)	6 (20%)	1 (8%)
	Patient’s questions are answered	9 (28%)	3 (11%)	2 (13%)
	No action necessary	4 (14%)	4 (14%)	1 (8%)
	Advice	3 (9%)	2 (5%)	2 (13%)
	Referral	1 (2%)	–	1 (8%)

## Discussion

The current study investigated the feasibility of RetinoQuest in clinical practice. Earlier studies have shown that computer-assisted data collection on HRQoL data is feasible in daily clinical practice [4, 5, 12–17, 19, 22, 29]. In line with the findings of de Bree et al. [14], we showed that RetinoQuest is a feasible e-health application to monitor HRQoL in Rb survivors in clinical practice. Ninety percent of the participants (*n* = 96) stated that the system was fast and easy to use. Furthermore, the system was rated with an overall satisfaction rate of 8.0 (scale 1–10).

The use of PROMs in pediatrics is scarce [16, 22, 24], while children with a chronic disease are at a greater risk of quality-of-life problems than a healthy reference group [3, 30]. Since RetinoQuest is developed for survivors of different age groups, the system uses three different validated questionnaires depending on the age group: the SDQ parent 4-10, SDQ 11-18, and the GHQ-28 [25–28]. Therefore, participants were divided into three evaluation groups: parent participants, adolescent participants, and adult participants. The parents of the children between 4 and 10 years were most often satisfied with the system. Meanwhile, adolescents rated the questionnaire the highest. The majority of this group (28 out of 38 respondents) stated that the questions were clear. The majority of the adolescent participants (63%) stated there were no redundant questions. A large group of the adult participants (41%) were dissatisfied with questions about severe depression, suicide, and death. Because GHQ-28 is a validated questionnaire, we chose not to omit or change items of this questionnaire. Currently, we inform the survivors and their parents about the fact that RetinoQuest includes a wide variety of questions (also regarding depression) due to the large heterogeneity in the survivor group.

Special attention was given to the visibility of RetinoQuest because many retinoblastoma survivors are visually impaired [31]. Questions were presented on a large screen in clear, large characters with bright colors. The majority (88%) of participants were able to read the questions adequately. Four blind adult participants were unable to read the screen and were assisted by a trained staff member who read out the questions. These findings indicate that computer-assisted data collection on HRQoL data should be tailored to the target group.

RetinoQuest facilitated the medical team in starting and having a structured conversation about the psychosocial condition and HRQoL of a patient, in addition to the medical follow-up. Although this study had a cross-sectional design, our clinical experience is that RetinoQuest also helps to provide clear insight in how specific problems improve or deteriorate over time. Podmore et al. [32] found that if clinicians rely on patients to initiate a discussion about psychosocial issues, patients’ problems may go unaddressed. Many patients do not spontaneously confess to doctors that they are suffering from psychosocial difficulties during consultation [17, 33].

From the structured interviews with the two HCPs at the end of the study, it was clear that RetinoQuest supports HCPs to communicate relevant psychosocial issues with survivors and their families. The interviews also revealed that HCPs appreciate the use of RetinoQuest in clinical practice. The discussion with survivors about their well-being profile is efficient and usually does not prolong the consultation time, which is in line with the study from Engelen et al. [23]. However, when problems are detected, it does take extra time to adequately support the patient, and it was emphasized that special psychological care should be available when necessary. The ophthalmologists’ observations confirmed that the PROMs included in RetinoQuest are satisfactory for clinical use, except for the questions about depression and suicide. Furthermore, they had the impression that the well-being profile matches the psychosocial situation of patient. The main flaw in the use of RetinoQuest was that the HCPs did not always have access to the well-being profile due to technical or logistical issues.

In the present study, the ophthalmologists used RetinoQuest in 80% of the visits to discuss the quality-of-life issues with the Rb survivors. Other authors found [14, 22, 29] that survivors and HCPs are willing to discuss the results of PROMs in clinical practice and that the possibility of data collection is depending on the technical and logistical capabilities of the organization.

An essential factor for implementing systems as RetinoQuest in clinical practice is having dedicated manpower (e.g., staff members) responsible for this task in supporting survivors to use the system in the hospital. Web-based data collection has shown to be logistically feasible [24]. This would allow visually impaired survivors to administer the tests to their own vision aid at home, thereby ensuring that no survivors are missed in hospital. Furthermore, it would give the HCPs the opportunity to screen the results in advance. We are currently working on a web-based version of RetinoQuest, so survivors can complete the questionnaire at home before the clinical visit.

One of the limitations of the current study is that the added value of the use of RetinoQuest during consultation was only evaluated from the HCPs’ point of view. Future studies should also focus on the question if survivors feel that the program adds value to their consultation with the HCP. Furthermore, it should also be investigated if RetinoQuest increases referrals for supportive care or counseling at clinical psychology.

## Conclusions

This study reveals that RetinoQuest is a feasible e-health application to monitor HRQoL in retinoblastoma survivors in clinical practice. Survivors as well as ophthalmologists are satisfied with the system as a tool to improve communication on quality of life and psychosocial difficulties. However, the use of the system is dependent on a good logistic organization.

## Electronic supplementary material

ESM 1 (DOCX 25 kb)
