# Supplementary material for: Feasibility of RetinoQuest: e-health application to facilitate and improve additional care for retinoblastoma survivors
Source: J Cancer Surviv. 2017 Sep 25;11(6):683–90. doi: 10.1007/s11764-017-0642-z (PMC5671548; doi:10.1007/s11764-017-0642-z)
Supplement: Supplementary file 1 — (DOCX 25 kb) [file 11764_2017_642_MOESM1_ESM.docx]

Online supplementary methods

*Ethical consideration*

The study protocol was approved by the Medical Ethics Committee (METC) of VUMC (METC-nr: 2011/191) and all procedures performed involving human participants were in accordance with the ethical standards of the institutional and/or national research committee and with the 1964 Helsinki declaration and its later amendments or comparable ethical standards.

The current evaluation study was performed in agreement with recommendations of Dutch ethics committee (“Niet Wet Medisch Wetenschappelijk Onderzoek met mensen - plichtig”, Dutch for “None Medical research involving human subjects act - obligation”) with waiver of informed consent. Prior to the current study survivors and their parents have been informed per letter regarding the use of RetinoQuest in the outpatient clinic to support ophthalmologic consultation. They were also informed that this use will be evaluated through an evaluation form. Apart from this, when filling out RetinoQuest, survivors are asked to give a consent to data analysis for scientific purposes by answering (YES/NO) the following question in the software application: “The information obtained is also important for scientific research at our department. Your information will be processed anonymously and treated confidentially. Please indicate below if you give permission to use this information for research purposes.”

*RetinoQuest*

RetinoQuest consists of the currently available validated questionnaires for oncological survivors in combination with Rb-specific questions. For parents the Dutch version of the Strengths and Difficulties Questionnaire 4-10 (SDQ parent 4-10) and for adolescents the SDQ 11-16 was implemented in RetinoQuest [1-3]. The SDQ is a 25-item questionnaire to monitor psychosocial problems in 4-16 year olds, with 5 subscales: emotional symptoms (5 items), conduct problems (5 items), hyperactivity/inattention (5 items), peer relationships (5 items), prosocial behavior (5 items). The General Health Questionnaire (GHQ28) was implemented in RetinoQuest for adult retinoblastoma survivors [4], which is a 28-item questionnaire with 4 subscales: somatic symptoms (7 items), anxiety and insomnia (7 items), social dysfunction (7 items) and severe depression (7 items). These validated questionnaires were implemented in RetinoQuest, since both SDQ and GHQ28 are used in the Netherlands routinely for follow up of all childhood cancer survivors.

RetinoQuest contains Rb related topics recommended by a clinical expert group (ophthalmologists, a retinoblastoma-specialized psychologist, an ocularist, a clinical geneticist, a pediatric oncologist and retinoblastoma-specialized nurses) and research [5]. The comprehensibility of the questions was tested in a group of 25 healthy children aged 11 to 12 years in a primary school.

The software program is written using Delphi2007 [6]. The first question is the survivor identification number, which is checked against the Central Patient Index (CPI) for confirmation. Each question is presented in a full screen mode, i.e. only one question at a time. After answering a question, the next question is displayed automatically on the screen. To meet the requirements of the visually impaired, the text is displayed in Verdana with a 24 to 28-point font size, in bright colors in boxes and displayed on a 17-inch touchscreen. Data of SDQ and GHQ of consecutive visits are translated into PROMs. The data is processed in real-time and is presented on the HCPs’ computer screen in separate graphs for each domain of the well-being profile.

*Procedure*

For a period of one year all eligible survivors and their parents, who visited the outpatient clinic, and two ophthalmologists, were interviewed regarding the use of and the satisfaction with RetinoQuest at the end of each visit.

RetinoQuest is a touch screen computer program to monitor HRQoL of Rb survivors via PROMs targeting children (4-10 years) as evaluated by their parents (proxy measures), adolescents (11-18 years), and adults. The computer is situated in a separate quiet room to ensure privacy and participants were supervised by a trained staff member (researcher).

*Analysis*

Participants were asked to complete an 8-item survey on feasibility of and satisfaction with RetinoQuest. The variables were measured via ten-point Likert scale, e.g; strongly agree (10 points), somewhat agree (8 points), neutral (6 points), somewhat disagree (4 points), strongly disagree (2 points). The time needed to complete RetinoQuest was logged by the computer program. Data was extracted from RetinoQuest and frequencies of the values and percentage frequencies were calculated for each variable. These frequencies are summarized in frequency table (Table 2). Ophthalmologists were asked to complete the question “Was the well-being profile discussed?” after each visit. In case of a negative answer, they were requested for an explanation and in case of a positive answer they were asked for the result of the discussion (e.g. advice or referral). Both ophthalmologists evaluated the use of RetinoQuest at the end of the study period through structured interviews.

**References**

1. Goodman R. The Strengths and Difficulties Questionnaire: a research note. J Child Psychol Psychiatry. 1997;38(5):581-6.

2. van Widenfelt BM, Goedhart AW, Treffers PD, Goodman R. Dutch version of the Strengths and Difficulties Questionnaire (SDQ). Eur Child Adolesc Psychiatry. 2003;12(6):281-9. doi:10.1007/s00787-003-0341-3 [doi].

3. youthinmind. The Strengths and Difficulties Questionnaire (SDQ). Published 2012. [www.sdqinfo.com](http://www.sdqinfo.com). Accessed 2016, April 10.

4. Koeter MWJ, Ormel J. General Health Questionnaire. Nederlandse bewerking. Lisse: Swets, Test Services. 1991.

5. van Dijk J, Oostrom KJ, Huisman J, Moll AC, Cohen-Kettenis PT, Ringens PJ et al. Restrictions in daily life after retinoblastoma from the perspective of the survivors. Pediatr Blood Cancer. 2010;54(1):110-5. doi:10.1002/pbc.22230 [doi].

6. de Bree R, Verdonck-de Leeuw IM, Keizer AL, Houffelaar A, Leemans CR. Touch screen computer-assisted health-related quality of life and distress data collection in head and neck cancer patients. Clin Otolaryngol. 2008;33(2):138-42. doi:10.1111/j.1749-4486.2008.01676.x [doi].
